# Impact of Dietary Manganese on Intestinal Barrier and Inflammatory Response in Broilers Challenged with *Salmonella* Typhimurium

**DOI:** 10.3390/microorganisms8050757

**Published:** 2020-05-18

**Authors:** Huaiyong Zhang, Shuqin Pan, Keying Zhang, Joris Michiels, Qiufeng Zeng, Xuemei Ding, Jianping Wang, Huanwei Peng, Jie Bai, Yue Xuan, Zhuowei Su, Shiping Bai

**Affiliations:** 1 Key Laboratory of Animal Disease-Resistant Nutrition, Ministry of Education, Institute of Animal Nutrition, Sichuan Agricultural University, Chengdu 611130, China; Huaiyong.zhang@Ugent.be (H.Z.); SQPan19@gmail.com (S.P.); zkeying@sicau.edu.cn (K.Z.); zqf@sicau.edu.cn (Q.Z.); dingxuemei0306@163.com (X.D.); wangjianping1983@hotmail.com (J.W.); phw@sicau.edu.cn (H.P.); baibing1901_cn@163.com (J.B.); xuanyuede1007@hotmail.com (Y.X.); wzs698@126.com (Z.S.); 2 Laboratory for Animal Nutrition and Animal Product Quality, Department of Animal Sciences and Aquatic Ecology, Ghent University, 9000 Ghent, Belgium; Joris.Michiels@UGent.be

**Keywords:** Mn, intestine, *Salmonella*, cytokine, mitochondria, broiler

## Abstract

Growing concern for public health and food safety has prompted a special interest in developing nutritional strategies for removing waterborne and foodborne pathogens, including *Salmonella*. Strong links between manganese (Mn) and intestinal barrier or immune function hint that dietary Mn supplementation is likely to be a promising approach to limit the loads of pathogens in broilers. Here, we provide evidence that *Salmonella* Typhimurium (*S.* Typhimurium, 4 × 10^8^ CFUs) challenge-induced intestinal injury along with systemic Mn redistribution in broilers. Further examining of the effect of dietary Mn treatments (a basal diet plus additional 0, 40, or 100 mg Mn/kg for corresponding to Mn-deficient, control, or Mn-surfeit diet, respectively) on intestinal barrier and inflammation status of broilers infected with *S.* Typhimurium revealed that birds fed the control and Mn-surfeit diets exhibited improved intestinal tight junctions and microbiota composition. Even without *Salmonella* infection, dietary Mn deficiency alone increased intestinal permeability by impairing intestinal tight junctions. In addition, when fed the control and Mn-surfeit diets, birds showed decreased *Salmonella* burdens in cecal content and spleen, with a concomitant increase in inflammatory cytokine levels in spleen. Furthermore, the dietary Mn-supplementation-mediated induction of cytokine production was probably associated with the nuclear factor kappa-B (NF-κB)/hydrogen peroxide (H_2_O_2_) pathway, as judged by the enhanced manganese superoxide dismutase activity and the increased H_2_O_2_ level in mitochondria, together with the increased mRNA level of *NF-κB* in spleen. Ingenuity-pathway analysis indicated that acute-phase response pathways, T helper type 1 pathway, and dendritic cell maturation were significantly activated by the dietary Mn supplementation. Our data suggest that dietary Mn supplementation could enhance intestinal barrier and splenic inflammatory response to fight against *Salmonella* infection in broilers.

## 1. Introduction

*Salmonella* is one of the most common foodborne and waterborne pathogens, and threats global public health and food safety. Infection by *Salmonella* usually occurs as a result of the consumption of non-potable waters or contaminated foods, particularly poultry meats and eggs. Epidemiological studies from various countries have indicated that food products of poultry origin are among the most common vehicles for transmission of *Salmonella* [1,2]. Thus, the reduction of *Salmonella* prevalence in poultry is paramount to our continued ability to limit this infectious threat.

The immunological effects of *Salmonella* infection in birds have been thoroughly reported despite the absence of gross clinical signs (except for the infection with *Salmonella* serovars Gallinarum or Pullorum) [3,4]. The scope of this response is age-dependent and is more obvious in chickens up to 2 weeks of age than in adult birds [5]. The disruption of tight junctions (TJs), the first line of defense, results in increased permeability to luminal bacteria and permits bacteria to approach the lymphatics and bloodstream, and even further to spread to liver and spleen [6]. Infection of broilers by *Salmonella* Typhimurium (*S.* Typhimurium) was found to decrease the expression of *occludin* and *claudin-1*, both important TJ proteins, and subsequently perturbed the epithelial barrier [7]. Similarly, in vitro experiments infecting T84 intestinal epithelial cells with *S.* Typhimurium caused a disruption in TJ function and facilitated bacterial translocation [8]. Another characteristic feature of *Salmonella* infection is the activation of the innate immune system, resulting in the proliferation of immune cells such as macrophages, heterophils, granulocytes, and dendritic cells [9]. Meanwhile, inflammatory cytokines such as interleukin (IL)-1, IL-6, IL-17, and IL-22, together with interferon-γ (IFN-γ) are released after infection, either by epithelial cells, resident phagocytes, infiltrating phagocytes, and/or lymphocytes in the cecum [3,10,11]. Recent data from our laboratory demonstrates that a similar cytokine gene expression pattern can be recorded in spleen, thymus, and bursa of broilers [12]. Oral *S.* Typhimurium challenge also was found to change the cluster of differentiation 4 (CD^4+^) and CD^8+^ cells ratios and increase the gene expression of *IL-1β*, *IL-8*, *IFN-γ*, and *IL-18* in the spleens of hens, similar to the response in the cecum after oral infection with *Salmonella* [13]. These proinflammatory cytokines are polypeptides that exert pleiotropic actions, regulating gene expression, host defense reaction, and the immune response. One of the important functions is the increase of reactive oxygen species (ROS) to induce a respiratory burst and bacterial killing [14,15] through mitochondria and nicotinamide adenine dinucleotide (NADPH) oxidase in phagocytic cells. 

Manganese (Mn), as a heavy metal, has become a public health concern due to serious environmental contamination and neurotoxicity. It is also an essential trace element of normal metabolism in humans and animals. The roles that Mn plays in innate and adaptive immune responses are well established in broilers [12], rats [16], and humans [17]. For instance, adding Mn to a diet could increase the phagocytic ability of natural killer cells of rats [16]. Exposure of the human monocyte-derived macrophages to Mn^2+^ (25–100 mM) alone or in combination with lipopolysaccharides caused dose-dependent increases in *IL-1β*, *IL-6*, *IL-8*, *IFN-γ*, and *tumor necrosis factor-α* (*TNF-α*) mRNA levels [18]. Dietary Mn added at high levels (600, 900, and 1800 mg Mn/kg diet) has been shown to upregulate mRNA levels of *nuclear factor-κB* (*NF-κB*), *cyclooxygenase-2* (*COX-2*), and *TNF-α* in chicken testes [19]. The data from our previous studies show that the diet with non-toxic high dose of Mn (400 vs. 40 mg Mn/kg of diet) also increased *IFN-γ* and *IL-6* mRNA expression in spleens of *Salmonella*-inoculated broilers at 2 days post inoculation (DPI) [12]. Further, data from human monocyte-derived macrophages [18] and chickens [19] reveal that one of the typical proposed mechanisms by which Mn increases production of inflammatory cytokines is mediated by the NF-κB pathway, which is a central regulator of the innate immune response upon *Salmonella* infection [20]. In addition to directly mediating activation of proinflammatory cytokines, NF-κB is also reported to transcriptionally upregulate the expression of *sod2*, a gene that encodes manganese superoxide dismutase (MnSOD), in murine embryonic fibroblasts (MEFs) [21,22]. This enzyme plays a key role in catalyzing the dismutation of superoxide anion into hydrogen peroxide (H_2_O_2_) and oxygen [23]. NF-κB-mediated activation of *sod2* expression thus probably ensures mitochondrial integrity of MEFs by scavenging ROS generated in the mitochondria during inflammatory responses [21,22]. Moreover, the production of H_2_O_2_ induced by MnSOD during inflammatory episodes has also been implicated as second messenger which in turn further exacerbates the transcription of various inflammatory mediators [18,24]. At this point, inducing H_2_O_2_ production by macrophages was found to upregulate the mRNA levels of inflammatory cytokines *IL-1β*, *IL-8*, *IFN-γ*, and *TNF-α* in human macrophages and neutrophils [18,25]. The review by Leto et al. (2009) also provides similar evidence saying that H_2_O_2_ could promote the transcription of NF-κB and prolong the production of inflammatory factors IL-1 and IL-6 [26]. Furthermore, a series of experimental studies had shown that dietary supplementation of a non-cytotoxic dose of Mn could up-regulate *sod2* mRNA level in chickens [27,28], illustrating that increases of H_2_O_2_ by dietary Mn-induced MnSOD activity probably act as intracellular messenger to upregulate the production of proinflammatory cytokines for fighting bacterial infections. 

Based on the above findings, we hypothesized that Mn added into chickens’ diets might improve intestinal integrity and enhance innate immunity towards *Salmonella* infection via NF-κB/H_2_O_2_-mediated inflammatory cytokine production to limit *Salmonella* count. With this aim, the objectives of the following studies were to determine the influence of dietary Mn alteration on intestinal morphology, MnSOD activity, and the content of mitochondrional ROS and H_2_O_2_, as well as inflammatory cytokines in broilers following oral *S.* Typhimurium challenge.

## 2. Materials and Methods

All of the procedures were conducted under the guidelines of the Animal Health and Care Committee in Sichuan Agricultural University (approval No. SAU-13-147).

### 2.1. Bacterial Culture

*S.* Typhimurium (SC0906) strain with spontaneous amikacin and neomycin resistance [27] was obtained from Veterinary Laboratory, Sichuan Agricultural University (Ya’an, China). The bacterium was cultured at 37 °C in a Luria-Bertani (LB; 10 g/L tryptone, 5 g/L yeast extract, and 10 g/L NaCl, pH 7.0–7.4) broth (Difco, Franklin Lakes, NJ, USA) for 10 hours with shaking before being used for the challenge. The number of colony-forming units (CFUs) of *S.* Typhimurium was determined by plating serial dilutions on count agar plates and 4 × 10^8^ CFU was used in this study.

### 2.2. Animals and Experimental Design

#### 2.2.1. Animals, Housing Conditions, and Diets

The chicks were obtained from a *Salmonella*-free flock as confirmed by the concentrations of serum immunoglobulin G using a commercial chicken ELISA kit (Sun Biomedical Technology Co. Ltd., Beijing, China). The birds which were seronegative for antibodies against *Salmonella* were reared in plastic-coated stainless-steel cages of 200 × 95 × 50 cm (length × width × height), located in an environmentally-controlled biosafety level-2 building. During the first week, the chickens were kept at 32 °C, which was reduced by 2 °C weekly, until the final temperature of 25 °C with a 30–40% relative humidity. Birds were maintained with 12 hours of light each day and allowed *ad libitum* access to diets and tap water containing no detectable Mn (<20 μg/L). The basal diet (Table S1) was formulated to meet the requirements recommended by the National Research Council (NRC, 1994) [29] except for Mn, and did not contain any antimicrobial agent. For dietary Mn treatments, the birds were fed one of three Mn-defined diets based on the NRC (1994); Mn-deficient, adequate (control), or Mn-surfeit diets. The Mn-deficient diet referred to the basal diet with no additional Mn, whereas the Mn control diet was defined as the basal diet plus additional 40 mg Mn/kg, and the Mn-surfeit diet corresponded to the basal diet plus additional 100 mg Mn/kg. The actual Mn concentrations of the diets were 25.6 ± 0.58 mg/kg Mn (Mn-deficient diet), 64.3 ± 0.16 mg/kg Mn (control diet), and 121.4 ± 1.34 mg/kg Mn (Mn-surfeit diet), respectively. All Mn levels used in this study did not exceed the Mn-toxicity dose of chicken based on previous report [30]. 

#### 2.2.2. Experiment 1 Design

To evaluate the effect of *Salmonella* challenge on the immune and Mn status, 144 chicks were allocated to 2 treatment groups with 6 replicate pens (12 birds/pen), and all birds were fed the control diets (the basal diet plus additional 40 mg Mn/kg) for 14 days. At 7 days-of-age, broilers were orally inoculated with *S*. Typhimurium at a dose of 4 × 10^8^ CFUs or isopycnic phosphate buffer saline (PBS) by crop inoculation as previously described [12]. Six birds of each subgroup were selected at 7 DPI. Blood was taken via jugular vein, followed by immediate excision of spleen, thymus, and bursa. Theses organs were weighed and stored at −80 °C pending analysis. Finally, jejunal mucosa was obtained for TJ expression analysis. At 7 DPI, another six chickens per treatment were selected and weighed for the intestinal permeability assay.

#### 2.2.3. Experiment 2 Design

To determine whether dietary Mn can alter the intestinal barrier, including TJs, and microbiome composition 216 chicks were firstly co-housed and fed the control diet for 4 days to ensure uniform and normalized microbiota composition. Subsequently birds were divided into three groups with 6 replicates (12 birds each) and fed either the Mn-deficient, the control, or the Mn-surfeit diets. All birds were orally inoculated with 4 × 10^8^ CFUs of *S.* Typhimurium at 7 days-of-age. Six birds of each subgroup were necropsied at 7 DPI. Mid-jejunum (about 1 cm) was obtained for hematoxylin and eosin (H&E) staining, mucosa and digesta of jejuna were collected for TJ expression and microbiota analysis, respectively. Also, another six chickens per treatment were selected and weighed for the intestinal permeability assay at 7 DPI.

#### 2.2.4. Experiment 3 Design

The aim of this experiment was to further examine the impact of dietary Mn on *Salmonella* burden and inflammatory response. Therefore, 216 chicks were divided into three groups (6 replicate pens; 12 birds/pen) and fed either the Mn-deficient, the control, or the Mn-surfeit diets. All birds were orally inoculated with 4 × 10^8^ CFUs of *S.* Typhimurium at 7 days-of-age. One chicken in each group was selected randomly at 7 DPI. Blood, spleen, thymus, bursa, and cecal content were collected and then stored at −80 °C until required.

#### 2.2.5. Experiment 4 Design

The objective of this trial was to examine the effect of Mn deficiency alone, without *S*. Typhimurium treatment, on intestinal barrier and inflammation status. For this purpose, 144 chicks were randomly divided into 2 treatment groups with 6 replicate pens (12 chicks/pen) and fed either the deficient or the control diets. At 14 days-of-age, six birds of each subgroup were selected. Blood was obtained for Mn and cytokine analysis. Mid-jejunum (about 1 cm) was obtained for H&E staining. Spleen, thymus, and bursa were excised and weighed immediately, followed by storage at −80 °C until analysis. Another six chickens per treatment were selected and weighed for the intestinal permeability assay at 14 days.

### 2.3. Intestinal Permeability Assay

At 7 DPI (14 days-of-age), one bird with similar body weight in each pen was selected, following a fasting period of 2 hours. Birds were given an oral gavage of 4.16 mg/kg fluorescein isothiocyanate (FITC)-dextran (4 kDa, FD4; Sigma, St. Louis, MO, USA), an indicator to examine barrier functions. Blood was obtained, 2.5 hours later, via jugular vein for collecting serum, and subsequently the concentration of FITC-dextran in the serum was measured at 485 nm excitation and 528 nm emission (BioTek Instruments, Winooski, VT, USA). The FITC-dextran concentrations were determined from standard curves generated by the serial dilution of FITC-dextran.

### 2.4. Mn Content Analysis

Mn contents in the diets and tissues were determined by atomic absorbance spectrophotometry (PerkinElmer AA800; PerkinElmer Inc., Spokane, WA, USA). Briefly, diets were ashed in a muffle furnace at 600 °C for 24 hours. Ashes, serum, liver, spleen, thymus, and bursa samples were digested by nitric acid using microwave digestion (Mars 5; CEM Corporation, Matthews, NC, USA), and then determined. A bovine liver standard (National Institute of Standards and Technology, Gaithersburg, MD, USA) was included in all analyses to verify the determination accuracy.

### 2.5. Intestinal Histomorphometry 

Specimens were fixed in 10% buffered neutral formalin, processed for paraffin wax sectioning into approximately 5-μm-thick pieces, and stained with H&E for light microscopy. The photomicrographs (Nikon Eclipse TS100; Nikon Corporation, Tokyo, Japan) were obtained to measure villus height and crypt depth using Image J (National Institutes of Health) at 100× magnification.

### 2.6. Quantitation of Serum Cytokines

The inflammatory cytokines contents including IL-1, IL-6, and TNF-α in serum were determined with commercial ELISA kits specific for chicken (Sun Biomedical Technology Co. Ltd., Beijing, China) according to the manufacturer’s instructions. All samples were tested in triplicate within each assay.

### 2.7. Quantitation of mRNA Using Real-Time PCR

The total RNA was isolated from frozen jejunum, spleen, thymus, and bursa, and then the concentration was determined using Nanodrop 2000 (Thermo Scientific, Waltham, MA, USA). Following purification, RNA (approximately 200 ng) was used as template for reverse transcriptase reactions using a reverse transcript kit (Takara, Japan). The resulting cDNA was immediately profiled for mRNA expression using a 7900 Fast Real-Time PCR System (Applied Biosystems, Foster City, CA, USA). *Glyceraldehyde-3-phosphate dehydrogenase* (*GAPDH*) and *β-actin* were selected as the reference genes, and relative gene expression was calculated as previously described [31]. Primer sequences for all genes are provided in Table S2.

### 2.8. Intestinal Microbial Population 

Bacterial DNA was isolated from jejunal content using stool DNA extraction kit (Omega Bio-Tek, Doraville, CA, USA) following the manufacturer’s instructions. The purity and concentration of DNA in each sample were measured by Nanodrop 2000. The populations of total bacteria, *Lactobacillus*, *Enterococcus*, *Salmonella*, *Bifidobacterium*, *Escherichia coli*, *Enterobacter*, and *Clostridium* were determined by q-PCR as described by Rezaei et al. (2015) [32]. The primers used for q-PCR were obtained from published works [32] and are listed in Table S3. Standard curves were prepared with serial dilutions of PCR products from pure cultures of the different bacterial groups, and copies per sample were calculated using Ct values and standard curves.

### 2.9. Enumeration of Salmonella

*Salmonella* counts were estimated using a standard plating method. Briefly, the collected cecal contents and spleens were processed upon arrival the day following collection. One gram of sample was suspended in 9 mL of 0.9% saline solution. The solution was diluted ten-fold to obtain dilutions of 1:10, 1:100, and 1:1000 and 100 μL of each dilution was pre-enriched for 24 hours at 37 °C in tetrathionate broth. A portion (100 μL) of the enriched culture was then plated in duplicate on selective brilliant green agar (BGA) supplemented with novobiocin (50 μg/mL). For BGA, 10.0 g of lactose, 10.0 g of sucrose, 5.0 g of casein peptone, 5.0 g of animal tissue peptone, 5.0 g of gelatin peptone, 5.0 g of NaCl, 3.0 g of yeast extract, 1.0 g of Na_2_HPO_4_, 0.09 g of phenol red, 0.52 g of KH_2_PO_4_, 4.7 g of brilliant green, and 12.0 g of agar were added to 1 L deionized water (pH 7.0–7.2). All plates were incubated for 24 h at 37 °C. Organisms giving typical small, smooth, dew-drop-like colonies with a pink background on BGS were used for initial judgement of *Salmonella*. Also, these colonies were further identified using the Microgen *Salmonella* Latex Agglutination kit (Microgen Bioproducts, Ltd., Surrey, UK) according to manufacturers’ instructions. Beyond that, the counts were calculated for 1 g of sample and converted into logarithm form. All microbiological analyses were performed in duplicate and the results were expressed as log_10_ CFU/g tissue.

### 2.10. Mitochondrion Isolation and MnSOD, ROS, and H_2_O_2_ Detection

Mitochondria were isolated from spleen, thymus, and bursa according to the method as previously described [33]. The quality of mitochondrial isolation was evaluated by calculating respiratory control ratios, which provide valuable information on the membrane integrity of isolated mitochondria [34]. Subsequently, dichlorofluorescein diacetate, an oxidation-sensitive dye, was employed to measure the mitochondrial ROS concentration using a spectrophotometer (excitation: 485 nm; emission: 528 nm). A spectrophotometric method based on the measurement of the absorbance of a solution in presence of horseradish peroxidase was used to detect the mitochondrial H_2_O_2_ concentration at 570 nm (BioTek Instruments, Winooski, VT, USA). Besides, approximately 0.5 g of spleen, thymus, or bursa was homogenized in ice-cold 0.9% sodium chloride buffer (*w*/*v*, 1:9) and then centrifuged at 1200× *g* for 10 min to obtain the supernatant. Subsequently the supernatant was used to determine MnSOD activity based on the hydroxylamine method. All procedures were performed at 4 °C, and all kits were obtained from Nanjing Jiancheng Bioengineering Institute (Nanjing, China). 

### 2.11. Ingenuity-Pathway Analysis (IPA)

IPA was done in order to identify the major biologic pathways of graded dietary Mn supplementation and inflammatory responses in broilers challenged with *S.* Typhimurium. The list of genes and the corresponding expression levels, represented as the log2 ratios, were uploaded within the IPA database and analysis performed (Ingenuity Systems^®^, http://www.ingenuity.com), which is a web-based large-scale causal network derived from the Ingenuity Knowledge Base that enables the visualization, discovery, and analysis of molecular interaction networks within gene expression profiles. Subsequently, Canonical Pathways Analysis that identified the pathways from the IPA library of canonical pathways were carried out, and functional pathways or networks with the highest confidence scores were determined. A *p* value was calculated using Fischer’s exact test to determine the probability that the association between the genes in the data set or the pathway is due to chance alone.

### 2.12. Statistical Analyses 

Statistical analysis of all data was performed using SAS 9.2 (SAS Institute, Cary, NC, USA). Statistical power of 0.80 (80%) was obtained in this study when the minimally-detectable effect size was 1.0 and the significance level was 0.05. Data were checked for normal distribution and equal variance. For all variables, except villus height and crypt depth of intestine, normal distribution was confirmed. Subsequently, differences between two groups were evaluated using a two-tailed unpaired *t*-test or the Mann−Whitney *U* test for normally or non-normally distributed datasets, respectively. The comparisons of more than two groups were performed with one-way analysis of variance (ANOVA) for normal distribution data with Tukey’s multiple comparison post-hoc test. In addition, a Kruskal−Wallis test followed by Dunn’s multiple comparisons was used for non-normal distribution data. Statistical significance was identified at *p* < 0.05.

## 3. Results

### 3.1. Salmonella Challenge Induces Splenomegaly and Mn Redistribution 

**Figure 1:**
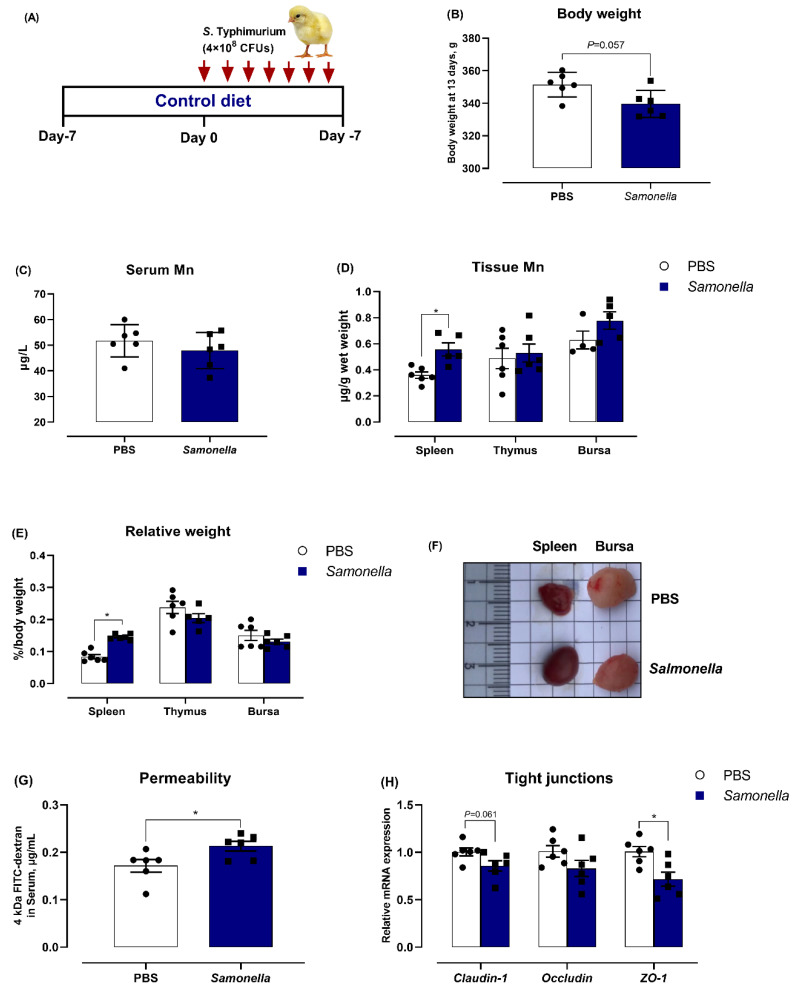
Oral *S*. Typhimurium infection induces Mn redistribution and splenomegaly and impairs the intestinal barrier. (**A**) Schematic presentation of the *S*. Typhimurium-challenge model. Birds were fed the control diets (the basal diet plus additional 40 mg Mn/kg) for 14 days and challenged with *S*. Typhimurium (4 × 10^8^ CFUs) or phosphate buffer saline (PBS) at 7 days-of-age. (**B**) Body weight changes were assessed. Levels of Mn were measured in (**C**) serum and (**D**) spleen, thymus, and bursa. (**E**) and (**F**), the relative weight of spleen, thymus and bursa were examined. The intestinal barrier was evaluated by permeability using of (**G**) fluorescein isothiocyanate (FITC)-dextran and (**H**) the mRNA level of tight junctions, including claudin-1, occludin, and zonula occludens-1 (ZO-1) in jejunal mucosa. Data represent means with standard deviation. * *p* < 0.05.

We explored the role of Mn during *Salmonella* infection by first investigating whether the *Salmonella* challenge altered the immune and Mn status. Birds were fed with the control diets for 7 days, followed by oral inoculation with *S*. Typhimurium or PBS (Figure 1A). As shown in Figure 1B, *S*. Typhimurium challenge was intended to reduce body weight of birds at 7 DPI (*p* = 0.057). The birds treated with *S*. typhimurium exhibited a slight decrease in serum Mn content and a 54.7%, 8.6%, and 23.8% increase in Mn level of spleen, thymus, and bursa when compared with the PBS-treated birds, respectively (Figure 1C,D). As compared to the PBS group, the relative weight of spleen was increased by 73.3% in the oral *S*. Typhimurium challenge group, but the *Salmonella* challenge did not affect the relative weights of thymus and bursa (Figure 1E,F). With regard to intestinal barrier, *S*. Typhimurium infection significantly increased intestinal permeability, shown by higher serum FITC-dextran concentrations (Figure 1G), together with downregulating the mRNA levels of jejunal TJs, especially ZO-1 and claudin-1 (Figure 1H). These data suggest that *S*. Typhimurium exposure induces intestinal injury and the redistribution of Mn in broilers at 7 DPI.

### 3.2. Dietary Mn Supplementation Improves Intestinal Integrity

**Figure 2:**
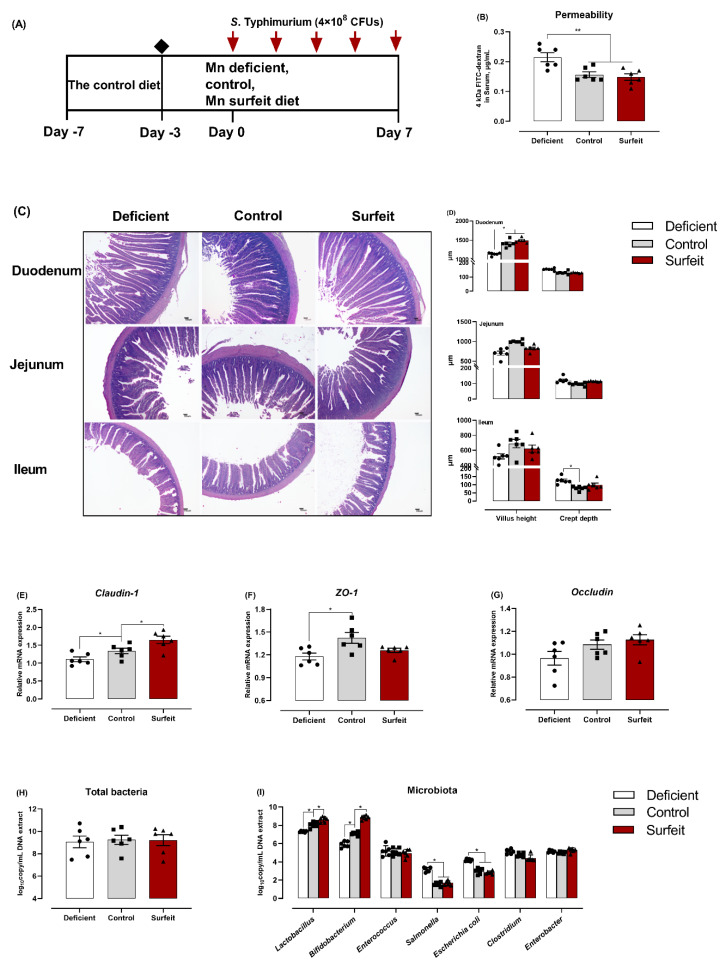
Dietary Mn supplementation improves intestinal barrier and intestinal microbiota of birds infected with *S*. Typhimurium. (**A**) Schematic presentation of dietary Mn treatments. One-day-old broilers were co-housed and fed the control diet (the basal diet plus additional 40 mg/kg Mn) for 4 days and then fed with either the Mn-deficient, the control, or the Mn-surfeit diets (the basal diet plus additional 0, 40, or 100 mg Mn/kg, respectively), for a further 10 days. All birds were orally inoculated with 4 × 10^8^ CFUs of *S*. Typhimurium at 7 days-of-age. (**B**) The permeability was evaluated by fluorescein isothiocyanate (FITC)-dextran. (**C**) Representative hematoxylin/eosin (H&E) staining and (**D**) villus height and crypt depth were measured. Scale bar = 100 μm. (**E**–**G**) RT-PCR quantification of claudin-1, zonula occludens-1 (ZO-1), and occludin in jejunal mucosa. (**H**) and (**I**) The composition of the microbiota from the digesta of jejuna. Data represent means with standard deviation. * *p* < 0.05; ** *p* < 0.01.

The intestinal epithelium is often the first interface between the host, the resident microbiota, and pathogenic microorganisms as a physiological and immunological barrier [8]. Thus, we determined whether dietary Mn alterations can impact on the intestinal barrier, including TJs and microbiota composition. It is well established that the composition of microbiota can alter in response to a number of factors such as strain, age, changes in the environment, medication, health, and especially diet [35]. After ensuring a uniform and normalized microbiota composition through feeding the control diet for 4 days, the birds subsequently were divided into three groups and fed either the Mn-deficient, the control, or Mn-surfeit diets for a further 10 days. All birds were orally inoculated with *S*. Typhimurium at 7 days-of-age (Figure 2A). Notably, the serum FITC-dextran levels were significantly higher in the Mn-deficient birds than in both the control and the Mn-surfeit birds (Figure 2B). The outcomes of histological analysis showed that the duodenum villus height of birds fed with the control and Mn-surfeit diets were significantly higher (*p* < 0.05) than the Mn-deficient diet. The villus height and crypt depth in jejuna were not significantly affected by Mn treatments. Additionally, the control diet caused lower (*p* < 0.05) crypt depth of ileum than the Mn-deficient diet (Figure 2C,D). Measurements of key components of TJs showed the Mn-deficient diet notably declined the mRNA level of claudin-1, ZO-1, and occludin in jejunal mucosa (Figure 2E–G), in contrast, gene expression of claudin-1 was significantly increased in the control and Mn-surfeit groups compared to the Mn-deficient group, and the expression of ZO-1 of birds fed control diet was also higher (*p* < 0.05) than those of the Mn-deficient diet (Figure 2E,F).

We also examined the effect of dietary Mn on the gut microbiota by collecting the digesta of jejuna at the end of the Mn manipulation (Days 14). Dietary Mn did not change the total bacterial load of jejuna (Figure 2H), whereas both the control and the Mn-surfeit diets markedly increased the colonization of *Lactobacillus* and *Bifidobacterium*, but decreased the colonization of *Salmonella* and *Escherichia coli* when compared with the Mn-deficient diet (Figure 2I). Taken together, these results indicate that dietary Mn supplementation improves the intestinal barrier and microbiome composition of broilers infected with *S*. Typhimurium. 

### 3.3. Dietary Mn Supplementation Decreases the Number of Salmonella

**Figure 3:**
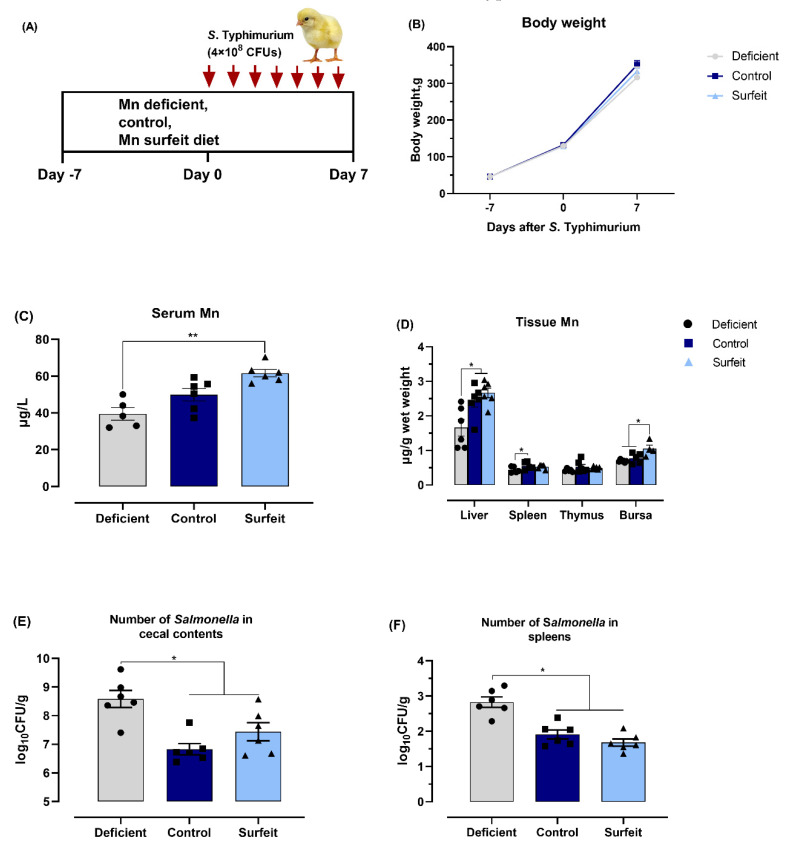
Dietary Mn supplementation increases tissue Mn levels and decreases the number of *Salmonella*. (**A**) Schematic presentation of study design where 1-day-old broilers fed with either the Mn-deficient, the control, or the Mn-surfeit diets (the basal diet plus additional 0, 40, or 100 mg Mn/kg, respectively), and up to 7 days following oral inoculation with *S.* Typhimurium till 14 days. (**B**) Body weight changes over time. Mn content in (**C**) serum and (**D**) tissues including liver, spleen, thymus, and bursa were determined. Bacterial burden in (**E**) cecal content and (**F**) spleen was estimated. Data represent means with standard deviation. * *p* < 0.05; ** *p* < 0.01.

Having established a condition that achieved dietary Mn deficiency and *S.* Typhimurium infection, we further fed broilers either the Mn-deficient, the control, or the Mn-surfeit diets for 14 days, and orally inoculated them with the *S*. Typhimurium at 7 days-of-age (Figure 3A) in order to examine the impact of dietary Mn on *Salmonella* burden and inflammatory responses. During the monitoring phases, broilers fed each of the diets displayed no difference in body weight (Figure 3B). With the graded supplementation of dietary Mn, birds exhibited correspondingly higher Mn deposition in serum, liver, spleen, and bursa, but not in thymus (Figure 3C,D). Moreover, *Salmonella* detection revealed that the Mn-deficient broilers exhibited significantly higher numbers of *Salmonella* in both cecal content and spleen, i.e., the control and the Mn-surfeit diets notably decreased *Salmonella* counts by 24.46% and 13.34% in cecal content, and 32.55% and 40.54% in spleen as compared with the Mn-deficient diet at 7 DPI, respectively (Figure 3E,F), suggesting the control and the Mn-surfeit diets reduced the *Salmonella* burden in birds treated with *S.* Typhimurium.

### 3.4. Dietary Mn Supplementation Promotes Splenic Inflammatory Response

**Figure 4:**
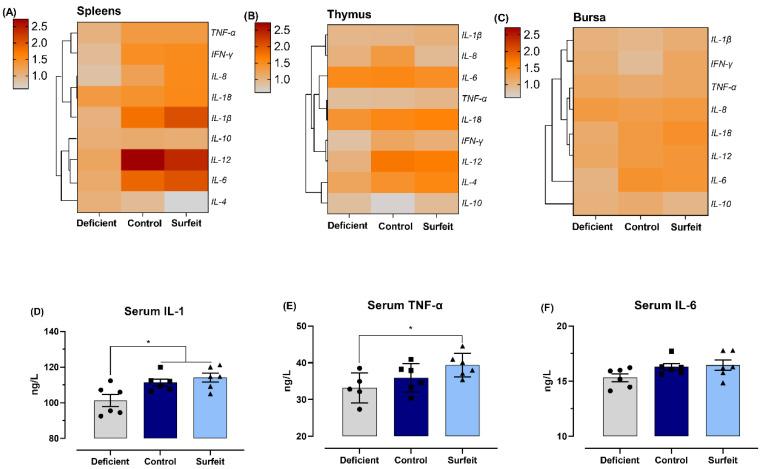
A heat map showing the mRNA expression of inflammatory cytokines in (**A**) spleen, (**B**) thymus, and (**C**) bursa from broilers fed the Mn-deficient, the control, or the Mn-surfeit diets with *S*. Typhimurium infection at 7 days-of-age. (**D**) IL-1, (**E**) IL-6, and (**F**) TNF-α content in the serum were determined. IL, interleukin; IFN, interferon; TNF, tumor necrosis factor. Data represent means with standard deviation. * *p* < 0.05.

Inflammatory response is a key cellular mechanism to fight again *Salmonella* infection, and one of potential mechanisms underlying Mn-promoting inflammatory cytokine production is mediated by the NF-κB/H_2_O_2_ pathway [18]. Therefore, we examined inflammatory cytokine expressions in spleen, thymus, and bursa, and the data are shown in Figure 4 and Table S4. In spleen, when compared with the Mn-deficient diet, the control diet increased IL-1β, IL-6, IL-8, IFN-γ, IL-12, and TNF-α mRNA levels, but no difference was found between control and surfeit diets. Further, the mRNA level of IL-4 was upregulated in birds fed the Mn-surfeit diet compared to broilers fed the Mn-deficient diet (Figure 4A). Concerning thymus and bursa, dietary Mn administration did not change the mRNA abundance of most inflammatory cytokines except that it significantly increased the IL-12 transcription in thymus and the IL-6 mRNA level in bursa (Figure 4B,C). In addition, the level of IL-1 was approximately 9.9% and 12.6% higher in serum of the control and the Mn-surfeit birds as compared to the Mn-deficient birds, respectively (Figure 4D), and the Mn-surfeit diet notably elevated serum TNF-α levels with respect to the Mn-deficient diet (Figure 4E). With the supplementation of dietary Mn, the concentration of IL-6 in the serum presented an increase, although it did not reach a significant level (Figure 4F).

**Figure 5:**
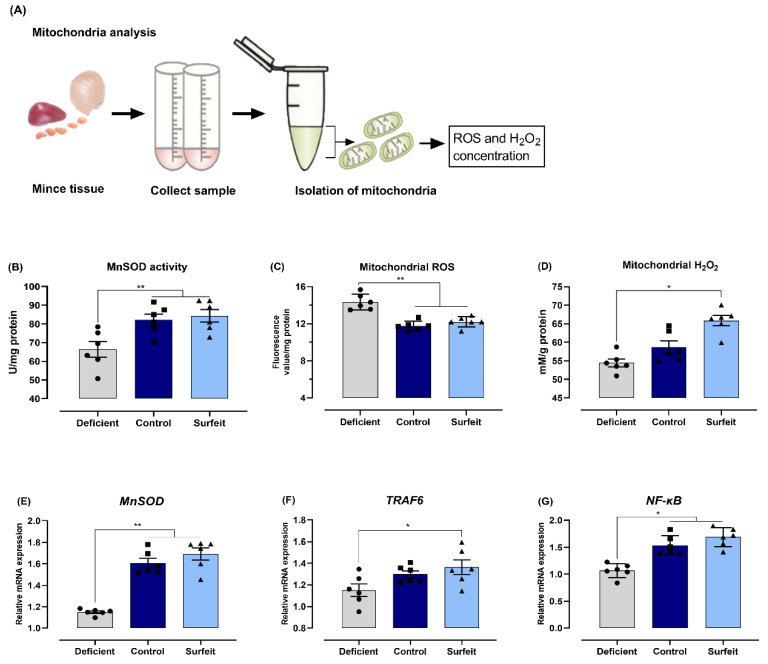
Mn supplementation associated with the activation of redox-sensitive signaling and downstream transcription factors. (**A**) Schematic diagram of extraction of mitochondria used in this study. (**B**) Manganese superoxide dismutase (MnSOD) activity, (**C**) reactive oxygen species (ROS) level, and (**D**) hydrogen peroxide (H_2_O_2_) concentration were determined in mitochondria isolated from spleens of birds challenged with *S*. Typhimurium. Meanwhile, mRNA expression of (**D**) MnSOD, (**E**) tumor necrosis factor receptor-associated factor 6 (TRAF6), and (**F**) nuclear factor-κB (NF-κB) in spleen were measured by RT-PCR. Data represent means with standard deviation. * *p* < 0.05; ** *p* < 0.01.

We also measured the MnSOD activity and the content of ROS and H_2_O_2_ in mitochondria, as well as the expression of key components of the NF-κB pathway. The results show that the MnSOD activity and the H_2_O_2_ content were significantly higher in the control and the Mn-surfeit birds than in the Mn-deficient birds, whereas lower ROS levels were observed in the control and the Mn-surfeit birds than in the Mn-deficient broilers in the mitochondria of spleen (Figure 5B–D). RT-PCR data showed substantial upregulation in the mRNA levels of MnSOD, TNF receptor-associated factor (TRAF) 6, and NF-κB in the spleens of the control and the Mn-surfeit birds when compared to birds fed the Mn-deficient diet (Figure 5E–G). However, in mitochondria of thymus and bursa, apart from the ROS concentration that was notably lower in the control and surfeit diets (Figure S1B), the MnSOD activity and H_2_O_2_ content, as well as the mRNA levels of MnSOD, TRAF6, and NF-κB were similar among the three groups (Figure S1). Collectively, these results demonstrate that dietary Mn supplementation promotes inflammatory responses in spleens of broilers against *S*. Typhimurium challenge, and this process might involve the upregulation of the NF-κB/H_2_O_2_ pathway.

### 3.5. Dietary Mn Interferes with Acute Inflammatory Reaction in the Spleen against S. Typhimurium Infection

**Figure 6:**
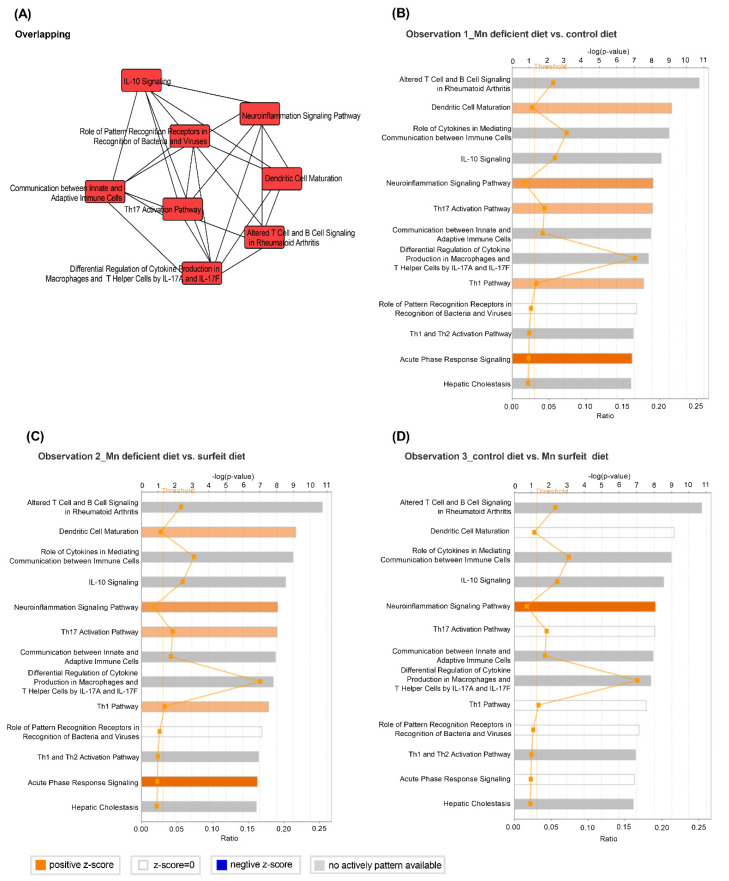
Ingenuity-pathway analysis (IPA) to identify activation of signaling pathways involved in dietary Mn and *S*. Typhimurium infection in broilers. (**A**) Top eight canonical pathways enriched by genes differentially expressed, and IPA of canonical pathways of differentially-altered genes expressed in (**B**) the Mn-deficient diet vs. the control diet; (**C**) the Mn-deficient diet vs. the Mn-supplemented diet, and (**D**) the control diet vs. the Mn-supplemented diet. A Fischer’s exact test was used to calculate a *p*-value.

To explore the potential biologic pathways underlying the dietary Mn modifying the inflammatory response in spleens of birds challenged with *S*. Typhimurium, pathway analysis of the inflammation-related genes was performed using the IPA software. The top pathways enriched for these genes included dendritic cell maturation, role of cytokines in mediating communication between immune cells, IL-10 signaling, the T helper type 1 (Th1) pathway, the Th17 activation pathway, and acute phase response signaling (Figure 6). Darker orange bars are pathways most activated in the IPA analysis. Thus, the most significantly-activated pathway was the Th1 pathway, the Th17 activation pathway, and acute phase response signaling (Figure 6B,C), indicating that supplementation of Mn mainly interfered with an acute inflammatory reaction in infected birds 7 DPI through the Th1 pathway, the Th17 pathway, or inducing dendritic cell maturation. However, a difference between control and surfeit groups’ effect on the acute inflammatory response was not observed (Figure 6D).

### 3.6. Dietary Mn Deficiency Itself Induces Intestinal Impairment but Does Not Affect Systemic Inflammation

**Figure 7:**
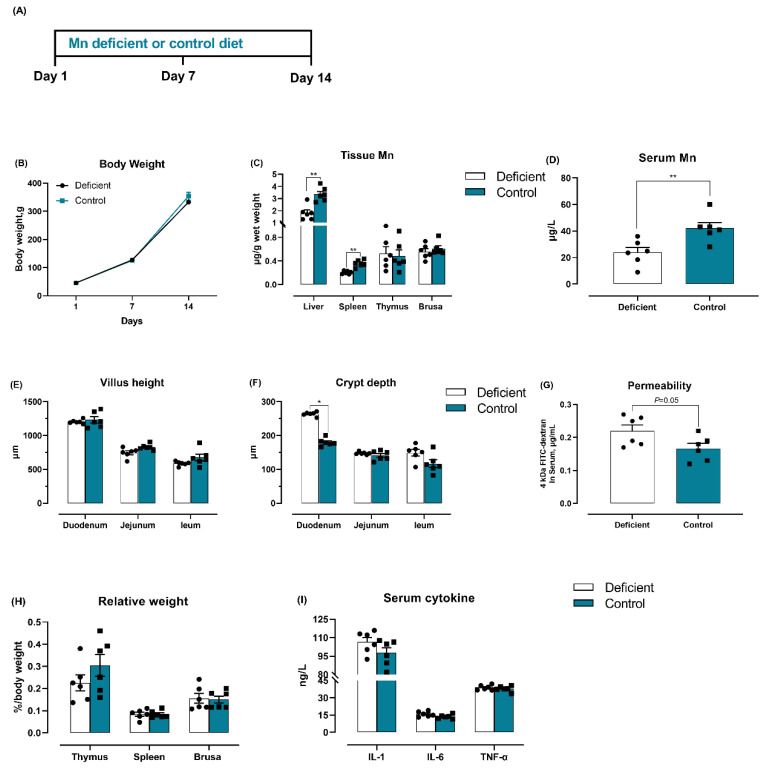
Dietary Mn deficiency itself induces intestinal impairment but did not affect systemic inflammation. (**A**) Schematic presentation of experimental design. Birds were fed with either the Mn-deficient or the control diet (the basal diet plus additional 0 and 40 mg Mn/kg, respectively). (**B**) Body weight was measured over the entire period. Levels of Mn were measured in (**C**) serum and (**D**) spleen, thymus, and bursa. The intestinal barrier was evaluated via (**E**) villus height and (**F**) crypt depth based on hematoxylin/eosin (H&E) staining, as well as (**G**) permeability using fluorescein isothiocyanate (FITC)-dextran. For systemic inflammation, (**H**) the relative weights of spleen, thymus, and bursa were examined, and (**I**) the serum inflammatory cytokines, including interleukin (IL)-1, IL-6, and tumor necrosis factor (TNF)-α were assessed. Data represent means with standard deviation. * *p* < 0.05; ** *p* < 0.01.

In our experimental paradigm, birds were fed the Mn-deficient diet two weeks prior to oral *S.* Typhimurium inoculation (Figure 3A). It is possible that dietary Mn deficiency itself could influence these parameters, thereby predisposing animals to a more severe appearance of *Salmonella* infection. We therefore examined the effect of Mn deficiency alone, without *S*. Typhimurium treatment, on intestinal barrier and inflammation status by feeding broilers with either the Mn-deficient or the control diet (Figure 7A). The Mn-deficient birds showed no significant change in body weight (Figure 7B), but showed a pronounced increased Mn level in liver, spleen, and serum as compared to the control birds (Figure 7C,D). No differences were observed in villus height of intestine among groups (Figure 7E), while the Mn-deficient diet significantly increased the crypt depth of duodenum (Figure 7F) and serum FITC-dextran level when compared with the control diet (Figure 7G). The results of inflammation status, evaluated by determining the relative weight of immune organs and serum cytokines, confirmed that dietary Mn level does not apparently impact the relative weight of thymus, spleen, and bursa (Figure 7H), and increased dietary Mn was not associated with marked changes in levels of serum IL-1, IL-6, and TNF-α (Figure 7I). These data indicate that dietary Mn deficiency itself impairs the intestinal barrier but does not induce release of inflammatory factors.

## 4. Discussion

Waterborne and foodborne pathogens, such as *Salmonella* have been, for decades, a major concern for public health and environment. Decreasing the *Salmonella* burden in chicken flocks, an important source of *Salmonella* infection, is a promising strategy to promote food safety. Our results provide some evidence that dietary Mn can maintain the integrity of the intestinal barrier and limit the infectious threat of *Salmonella* from animal foods.

The infection of *Salmonella* induces a productivity decline or many side effects in broilers [36,37]. Similarly, in our study, the deleterious effect of *S.* Typhimurium challenge was demonstrated by decreased body weight. In previous studies, *Salmonella* infection was found to induce pronounced splenomegaly of mice [38], and greater increases in bursa, thymus, and spleen weight of growing chicks [39], which also corresponds to the results of the current study. The increased relative weight of spleen indicates that these chicks underwent a greater systemic inflammatory response [39]. Meanwhile, we noticed that *Salmonella* infection was accompanied by Mn redistribution (serum Mn decreased and spleen Mn increased) herein, similar to the response in a mouse model of *Staphylococcus aureus* infection [40]. A possible explanation might be that Mn is related to systemic immune responses of broilers against *S.* Typhimurium challenge [12,41]. Based on this consideration we reasoned that altering systemic Mn homeostasis through dietary Mn supplementation could enhance the host’s immune response to *Salmonella* inoculation.

Another striking feature of *Salmonella* infection is its impact on the impairment of the intestinal barrier. Previous studies showed that *Salmonella* infection caused a disruption of TJ structure in broilers [7] and T84 intestinal epithelial cells [8]. Our results consistently demonstrated that the challenge with *S.* Typhimurium resulted in a downregulation of the expression of *claudin-1* and *ZO-1*, and an increase in intestinal permeability in broilers. Subsequently, alterations in dietary Mn confirmed that Mn deficiency increased the severity of *Salmonella* infection when compared to a Mn-adequate diet through increasing intestinal permeability and depressing gene expression of TJs. Even in the absence of *S.* Typhimurium treatment, birds fed with the Mn-deficient diet displayed a 1.32-fold greater permeability relative to those fed the control diet. In contrast, increased dietary Mn intake by broilers has been associated with marked improvements in the intestinal barrier. These results are congruent with previous outcomes in mice and indicate that Mn is necessary for proper maintenance of the intestinal barrier. For example, Mn provided protection against dextran sulfate sodium (DSS)-induced colon injury [42]. It was postulated that the positive effect of Mn on the intestinal barrier was mediated by MnSOD [23], which is in agreement with the efficacious protection of the small intestine from ionizing radiation damage through overexpression of MnSOD in mice [43]. Besides changes in TJs, the infection of *Salmonella* was also associated with an increase in *Enterobacteriaceae* in cecal microbiota in chickens [44], and influenced the composition of the ileum mucosa microbiota in pigs, reflected by a decrease in representatives of the generally-regarded-as-desirable genera (i.e., *Bifidobacterium* and *Lactobacillus*) [45]. Mice administered with MnCl_2_ (20 mg/kg body weight/day) by drinking water for 13 weeks also exhibited an apparent increase in the relative abundance of *Lactobacillus* and *Bifidobacterium* [46]. Excitingly, the present study clearly showed that supplementing Mn is effective in increasing the populations of *Lactobacillus* and *Bifidobacterium* and decreasing the populations of *Salmonella* and *Escherichia coli* in jejunal digesta of broilers following oral *S.* Typhimurium, indicating dietary Mn can optimize intestinal microbiota composition. Thus, adequate Mn is necessary for maintaining the intestinal barrier and provides protection against *Salmonella* infection.

The disruption of the intestinal epithelial barrier by the Mn-deficient diet is also reflected by higher *Salmonella* burden in cecal content and translocation into the spleen in our current study. Besides the induction of intestinal inflammatory cytokine release such as IL-1, IL-6, IL-17, or IFN-γ [4,11], *Salmonella* challenge was also found to trigger a similar cytokine gene expression in the spleen, thymus, and bursa of chickens [13,47]. In this study, we assessed whether dietary Mn alterations were associated with changes in the gene expressions of cytokines. We revealed that the control diet stimulated the expression of inflammatory cytokines in immune organs, especially the spleen, when compared with the Mn-deficient diet, which was consistent with our previous findings on the rapid increase of *IL-1β* and *IL-6* mRNA expression in spleens of *Salmonella*-infected broilers [12]. A similar result has also been reported where Mn markedly increased the production of inflammatory cytokines and chemokines in human monocyte-derived macrophages [18]. However, Choi and colleagues reported that a diet with adequate Mn notably depressed the expression of inflammatory cytokines *TNF-α*, *IL-6*, *IL-1β*, and *IL-10* when compared to the Mn-deficient diet in colon mucosa of DSS-treated mice [42]. Excess Mn exposure was also reported to inhibit or induce the expression of genes of proinflammatory cytokines in chickens [19,41], indicating that dietary Mn modifies inflammatory cytokines in a dose-dependent manner and/or is dependent on the experimental model. In the present study, the changes in cytokine profile of the blood partly reflected the fluctuation of splenic inflammation reactions in broilers treated with *S.* Typhimurium and dietary-different Mn. It needs to be emphasized that unlike the effects observed in *Salmonella*-treated birds, serum inflammatory cytokines IL-1, IL-6, and TNF-α were not altered by Mn itself. Thus, we speculate that the fact that dietary Mn promotes inflammatory response in the spleen might play a key role in the fight against *S.* Typhimurium challenge for broilers.

We applied the IPA program to explore the potential biologic pathways based on the list of differentially-expressed genes, and the data revealed that, in the current study, dietary Mn alteration is related to the Th1 activation pathway. We provided evidence that dietary Mn supplementation activates the Th1-cytokine response in the spleen to *S.* Typhimurium infection. Indeed, the remarkable induction of IL-1β, IL-6, IFN-γ, and IL-12 are well-known Th1 cytokines. In addition, the IPA results from this study demonstrate that dietary Mn supplementation is involved in dendritic cell maturation and acute-phase response pathways, which are active during inflammation and/or as a contribution to the immune response of chicken against *Salmonella* [4]. Other pathways were also enriched including IL-10 signaling, role of cytokines in mediating communication between immune cells signaling, and particularly the Th17 activation pathway. It seems that Th17-mediated signaling pathways may also play an important role in the effect of dietary Mn levels on the inflammatory response of spleen in broilers following *Salmonella* challenge [48]. 

It is well known that the NF-κB signaling pathway involves the regulation of cytokine and chemokine release in response to *Salmonella* infection [20], and the production of H_2_O_2_ induced by MnSOD during inflammatory episodes could be a second messenger that further exacerbates the transcription of various inflammatory mediators [18,24]. In the present study, we consistently found that dietary Mn supplementation enhanced MnSOD-mediated dismutation of superoxide anion into H_2_O_2_ in splenic mitochondria and upregulated the mRNA abundance of *TRAF6* and *NF-κB* in the spleen. TRAF6, an E3 ubiquitin, is regulated through intracellular homeostatic processes, such as oxidative stress [49]. Activated TRAF6 has the ability to catalyze the formation of Lys-63-linked polyubiquitin chains on itself and on other protein substrates to induce NF-κB activation [50]. Similarly to our findings, Mokgobu and colleagues confirmed that the Mn^2+^-mediated induction of cytokine production was associated with increased production of H_2_O_2_ and was completely attenuated by inclusion of the H_2_O_2_-scavenger dithiothreitol, and partially by inhibitors of NF-κB kinase [18]. Thus, these findings indicate that dietary Mn regulating splenic inflammatory response to fight against *Salmonella* might involve the NF-κB/H_2_O_2_-mediated signaling pathway.

Limitations of our study include that the data on dose-dependent effects of dietary Mn on immune status, especially in intestine, are lacking. Second, regarding the mechanisms underlining dietary Mn action on inflammatory cytokine, it is possible that Mn addition results in inflammatory changes in chickens following oral *S.* Typhimurium are through another pathway, such as the NF-κB/nitric oxide synthase-COX-2 pathway [19]. The third limitation is the statistical assessment. Unequal variances and non-normal distribution were observed in some data sets, which contributes to an insufficient number of samples. Therefore, we admit the possibility that some of our conclusions may include overestimation or underestimation of roles of dietary Mn in intestinal barrier and immune responses of broilers challenged with *S.* Typhimurium. Further experiments would be essential to exclude this possibility.

## 5. Conclusions

The present study demonstrates that dietary Mn supplementation is critical to the maintenance of the intestinal barrier and promotes splenic inflammatory response against *Salmonella* infection, and this possibly involves the NF-κB/H_2_O_2_-mediated signaling pathway. These results will bring a promising strategy for development in augmenting host-mediated nutritional immunity by exploiting Mn-dependent pathways to limit waterborne and foodborne pathogens.

## Supplementary Materials

The following are available online at https://www.mdpi.com/2076-2607/8/5/757/s1, Table S1. Composition and nutrient levels in the basal diets (dry matter basis); Table S2. The primers for quantitative real-time PCR; Table S3. Sequences of the primers used for the determination of the microbial population; Table S4. Effects of graded Mn on MnSOD and inflammatory cytokine expressions in spleen, thymus, and bursa of broilers following oral *S.* Typhimurium; Figure S1: Effect of dietary Mn alterations on the activation of redox-sensitive signaling and downstream transcription factors in thymus and bursa of birds challenged with *S.* Typhimurium.
