# Supplementary material for: Impact of Dietary Manganese on Intestinal Barrier and Inflammatory Response in Broilers Challenged with Salmonella Typhimurium
Source: Microorganisms. 2020 May 18;8(5):757. doi: 10.3390/microorganisms8050757 (PMC7285304; doi:10.3390/microorganisms8050757)
Supplement: Supplementary file 1 [file microorganisms-08-00757-s001.pdf]

**Table S1.** Composition and nutrient levels in the basal diets (dry matter basis)

| Ingredients | g/kg diet | Nutrient analysis | g/kg diet |
|---|---|---|---|
| Ground corn | 543.0 | AME/(kcal/kg) | 2950 |
| Soybean oil | 34.0 | CP | 210 |
| Soybean meal (44%) | 380.2 | Calcium | 10.1 |
| Lysine (98%) | 1.5 | Available Phosphorus | 4.5 |
| DL-Methionine | 2.5 | Lysine | 11.5 |
| Calcium carbonate | 11.4 | Methionine | 5.0 |
| Di-calcium Phosphate | 18.6 | Methionine and Cystine | 8.6 |
| Sodium chloride | 4.0 | Manganese (mg/kg)[3] | 25.62 |
| Choline chloride | 1.5 | | |
| Vitamin premix[1] | 0.3 | | |
| Mineral premix[2] | 3.0 | | |
| **Total** | 1000 | | |

[1] Provided per kilogram of diet: Vitamin A 8000 IU; Vitamin $D_3$ 1000 IU; Vitamin E 20 IU; Vitamin $K_3$ 0.5 mg; Pantothenic acid 10 mg; Niacin amide 35 mg; Folic acid 0.55 mg; Biotin 0.18 mg.

[2] Provided per kilogram of diet: Cu ($CuSO_4{\cdot}5H_2O$), 8 mg; Fe ($FeSO_4{\cdot}7H_2O$), 80 mg; Zn ($ZnSO_4{\cdot}7H_2O$), 70; Se ($NaSeO_3$), 0.3 mg; I (KI), 0.7 mg.

[3]Manganese concentration in the basal diet was analyzed by atomic absorption spectrophotometer.

AME, apparent metabolism energy; CP, crude protein;

**Table S2.** The primers for quantitative real-time PCR

| Gene | Gene ID | Primer | Sequence (5′-3′) | Product Size (bp) |
|---|---|---|---|---|
| *IL-1β* | NM_204524.1 | Reverse | gcatcaagggctacaagctc | 131 |
| | | Forward | caggcggtagaagatgaagc | |
| *IL-6* | NM_204628.1 | Reverse | ctcctcgccaatctgaagtc | 100 |
| | | Forward | ccctcacggtcttctccata | |
| *IL-8* | AJ009800 | Reverse | gcttgctaggggaaatgaag | 136 |
| | | Forward | ggaattaccagtttgctgctg | |
| *IL-12β* | NM_213571.1 | Reverse | cctgcctgtctgctaagacc | 82 |
| | | Forward | atcatttgcccattggagtc | |
| *IL-18* | NM_204608.1 | Reverse | tgaaatctggcagtggaatg | 144 |
| | | Forward | caaccattttcccatgctct | |
| *IFN-γ* | NM_205149.1 | Reverse | cagatgtagctgacggtgga | 98 |
| | | Forward | catcgaaacaatctggctca | |
| *TNF-α* | GU230788.1 | Reverse | gcccttcctgtaaccagatg | 71 |
| | | Forward | acacgacagccaagtcaacg | |
| *TRAF6* | XM_015287208.1 | Reverse | atggaagccaagccagagtt | 144 |
| | | Forward | acagcgcaccagaagggtat | |
| *NF-κB* | M86930.1 | Reverse | tcaacgcaggacctaaagacat | 162 |
| | | Forward | gcagatagccaagttcaggatg | |
| *MnSOD* | NM_204211.1 | Reverse | ttcctgacctgccttacgactat | 138 |
| | | Forward | ccagcgcctctttgtatttct | |
| *claudin-1* | NM_001013611.2 | Reverse | gtctttggtggcgtgatctt | 117 |
| | | Forward | tctggtgttaacgggtgtga | |
| *ZO-1* | XM_015278981.2 | Reverse | ggtcagccagatgtggattt | 81 |
| | | Forward | ccgaagcattccatcttcat | |
| *Occludin* | NM_205128.1 | Reverse | gctgagatggacagcatcaa | 97 |
| | | Forward | cctctgccacatcctggtat | |
| *β-actin* | NM_205518.1 | Reverse | gctacagcttcaccaccaca | 90 |
| | | Forward | tctcctgctcgaaatccagt | |
| *GAPDH* | NM_204305.1 | Reverse | tgggaagcttactggaatgg | 88 |
| | | Forward | cttggctggtttctccagac | |

*IL,* interleukin; *IFN,* interferon; *TNF,* tumor necrosis factor. *TRAF6*, TNF receptor-associated factor 6. *NF-κB*, nuclear transcription factor kappa B; *MnSOD*, manganese superoxide dismutase; *ZO-1*, zona occludens 1; *GAPDH*, glyceraldehyde-3-phosphate dehydrogenase.

**Table S3**. Sequences of the primers used for the determination of the microbial population

| Items | Primer | Sequence (5′-3′) |
| --- | --- | --- |
| Total bacteria | Reverse | gatccgcttgccttcgca |
| | Forward | catccagtgcaaacctaagag |
| *Lactobacillus* | Reverse | gatccgcttgccttcgca |
| | Forward | catccagtgcaaacctaagag |
| *Bifidobacterium* | Reverse | taagccatggactttcacacc |
| | Forward | gggtggtaatgccggatg |
| *Enterococcus* | Reverse | act cgttgtacttcccattgt |
| | Forward | cccttattgttagttgccatcatt |
| *Salmonella* | Reverse | aaacgttgaaaaactgagga |
| | Forward | tcgtcattccattacctacc |
| *Escherichia coli* | Reverse | agaacgctttgtggttaatcagga |
| | Forward | gtgtgatatctacccgcttcgc |
| *Clostridium* | Reverse | ccctttacacccagtaa |
| | Forward | gagtttgatcmtggctcag |
| *Enterobacter* | Reverse | ctctacgagactcaagcttgc |
| | Forward | cattgacgttacccgcag aagaagc |

**Table S4**. Effects of graded Mn on *MnSOD* and inflammatory cytokine expressions in spleen, thymus, and bursa of broilers following oral *S.* Typhimurium

| Gene | Tissue | Dietary Mn levels | | |
|---|---|---|---|---|
| | | Deficient diet (0 mg/kg) | Control diet (40 mg/kg) | Surfeit diet (100 mg/kg) |
| *TNF-α* | Spleen | 1.03±0.06$^{b}$ | 1.33±0.06$^{a}$ | 1.33±0.01$^{a}$ |
| *IFN-γ* | Spleen | 0.91±0.17$^{b}$ | 1.48±0.18$^{a}$ | 1.51±0.20$^{a}$ |
| *IL-8* | Spleen | 0.84±0.14$^{b}$ | 1.24±0.20$^{a}$ | 1.53±0.04$^{a}$ |
| *IL-18* | Spleen | 1.32±0.07 | 1.43±0.10 | 1.54±0.32 |
| *IL-1β* | Spleen | 1.03±0.24$^{c}$ | 1.78±0.33$^{b}$ | 2.16±0.14$^{a}$ |
| *IL-10* | Spleen | 1.07±0.20 | 1.11±0.17 | 1.09±0.29 |
| *IL-12* | Spleen | 1.19±0.11$^{b}$ | 2.72±0.56$^{a}$ | 2.38±0.62$^{a}$ |
| *IL-6* | Spleen | 1.12±0.25 | 1.87±0.59 | 2.03±0.90 |
| *IL-4* | Spleen | 1.05±0.48$^{a}$ | 0.91±0.25$^{ab}$ | 0.60±0.18$^{b}$ |
| *MnSOD* | Spleen | 1.15±0.03$^{b}$ | 1.61±0.11$^{a}$ | 1.69±0.14$^{a}$ |
| *TRAF6* | Spleen | 1.15±0.14$^{b}$ | 1.30±0.08$^{ab}$ | 1.37±0.16$^{a}$ |
| *NF-κB* | Spleen | 1.07±0.13$^{b}$ | 1.53±0.18$^{a}$ | 1.69±0.17$^{a}$ |
| *IL-1β* | Thymus | 0.97±0.03 | 0.98±0.08 | 1.05±0.14 |
| *IL-8* | Thymus | 1.04±0.03$^{b}$ | 1.33±0.25$^{a}$ | 0.92±0.08$^{b}$ |
| *IL-6* | Thymus | 1.52±0.04 | 1.55±0.14 | 1.48±0.09 |
| *TNF-α* | Thymus | 0.89±0.04 | 0.91±0.04 | 0.98±0.04 |
| *IL-18* | Thymus | 1.41±0.09 | 1.55±0.20 | 1.62±0.09 |
| *IFN-γ* | Thymus | 0.85±0.09 | 1.18±0.13 | 1.07±0.08 |
| *IL-12* | Thymus | 1.03±0.05$^{b}$ | 1.73±0.26$^{a}$ | 1.68±0.44$^{a}$ |
| *IL-4* | Thymus | 1.20±0.38 | 1.44±0.73 | 1.57±0.36 |
| *IL-10* | Thymus | 0.87±0.06 | 0.63±0.03 | 0.91±0.10 |
| *MnSOD* | Thymus | 1.37±0.12 | 1.44±0.20 | 1.48±0.18 |
| *TRAF6* | Thymus | 1.39±0.20 | 1.54±0.14 | 1.63±0.21 |
| *NF-κB* | Thymus | 1.19±0.13 | 1.27±0.11 | 1.22±0.08 |
| *IL-1β* | Bursa | 1.04±0.22 | 0.97±0.15 | 1.09±0.06 |
| *IFN-γ* | Bursa | 1.08±0.30 | 0.91±0.25 | 1.17±0.21 |
| *TNF-α* | Bursa | 1.18±0.06 | 1.11±0.04 | 1.18±0.05 |
| *IL-8* | Bursa | 1.33±1.73 | 1.28±1.01 | 1.34±0.65 |
| *IL-18* | Bursa | 1.12±0.30 | 1.33±0.17 | 1.45±0.04 |
| *IL-12* | Bursa | 1.16±4.11 | 1.33±3.12 | 1.38±0.31 |
| *IL-6* | Bursa | 1.01±0.62$^{b}$ | 1.43±1.09$^{a}$ | 1.39±0.35$^{ab}$ |
| *IL-10* | Bursa | 1.04±0.46 | 1.11±0.29 | 0.98±0.19 |
| *MnSOD* | Bursa | 1.17±0.08 | 1.22±0.10 | 1.23±0.09 |
| *TRAF6* | Bursa | 1.26±0.06 | 1.32±0.08 | 1.37±0.10 |
| *NF-κB* | Bursa | 1.31±0.14 | 1.34±0.05 | 1.47±0.11 |

Data represent means with standard deviation. $^{a,\ b}$ Mean values with different letters are significantly different (one-way ANOVA, $P<0.05$, Tukey's *post hoc* test).

*IL*, interleukin; *IFN,* interferon; *TNF,* tumor necrosis factor. *TRAF6*, TNF receptor-associated factor 6. *NF-κB*, nuclear transcription factor kappa B; *MnSOD*, manganese superoxide dismutase; *GAPDH*, glyceraldehyde-3-phosphate dehydrogenase.

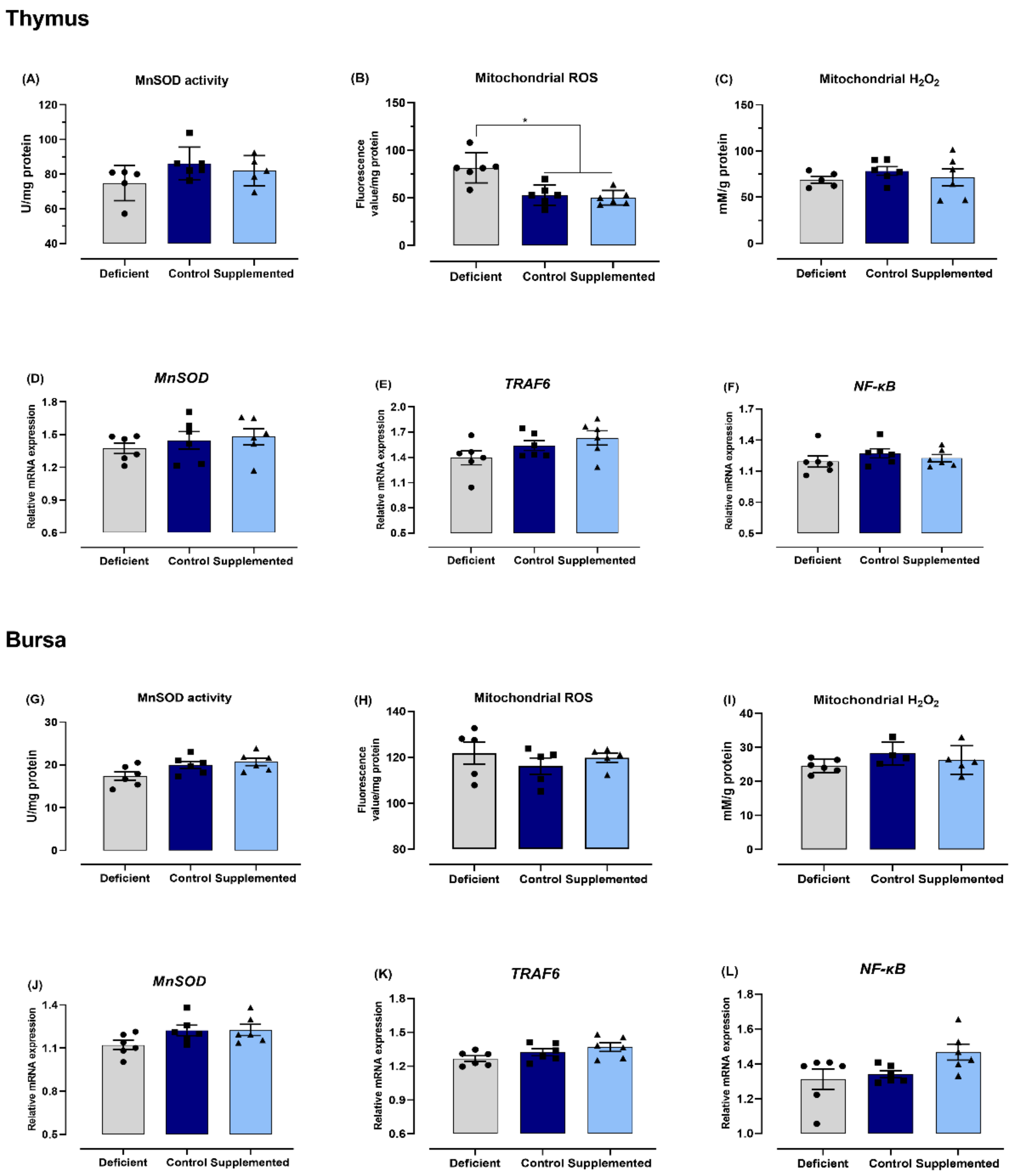


**Figure S1** Effect of dietary Mn alterations on the activation of redox-sensitive signaling and downstream transcription factors in thymus and bursa of birds challenged with *S.* Typhimurium. (A) and (G) Manganese superoxide dismutase (MnSOD) activity, (B) and (H) reactive oxygen species (ROS) level, and (C) and (I) hydrogen peroxide ($H_2O_2$) concentration were determined in mitochondria isolated from thymus or bursa, respectively. Meanwhile, mRNA expression of (D) *MnSOD*, (E) tumor necrosis factor receptor-associated factor 6 (*TRAF6*), and (F) nuclear factor kappa-B (*NF-κB*) in thymus or bursa were measured by RT-PCR, respectively. Data represent means with standard deviation. **P* < 0.05.
